# Use of fractional and microablative CO2 laser in cases of vulvar lichen sclerosus: a clinical-histological analysis

**DOI:** 10.1007/s10103-026-04923-3

**Published:** 2026-06-26

**Authors:** Dayane de Assis Pereira Hansen Cavalheiro, Ana Carolina Silva Chuery, Milvia Maria Simões e Silva Enokihara, Helio Amante Miot, Mauricio Fillippini, Neila Maria de Góis Speck

**Affiliations:** 1 Postgraduate Program of the Department of Gynecology, Federal University of São Paulo, São Paulo, Brazil; 2 Department of Gynecology, Paulista School of Medicine, Federal University of São Paulo, São Paulo, Brazil; 3 Department of Pathology, Paulista School of Medicine, Federal University of São Paulo, São Paulo, Brazil; 4 Department of Dermatology, Botucatu School of Medicine, Paulista State University, Botucatu, Brazil; 5 Department of Obstetrics and Gynecology, State Hospital of Republic of San Marino, San Marino, San Marino

**Keywords:** Vulvar lichen sclerosus, CO₂ laser, Fractional and microablative laser, Collagen fibers, Elastic fibers, Vulvar biopsy

## Abstract

Objective: To evaluate the clinical and histological response of patients with vulvar lichen sclerosus (VLS) following fractional and microablative CO₂ laser treatment. Methods: This interventional clinical study included 14 patients diagnosed with VLS. The participants underwent three CO₂ laser sessions with a monthly interval. Clinical assessments and biopsies were performed at baseline and six weeks after the final session. Primary outcomes were improvement in symptoms (Visual Analog Scale – VAS) and clinical signs (Global Aesthetic Improvement Scale – GAIS), associated with histological regression. Secondary outcomes included quality of life (Skindex-29) and specific histological parameters, such as hyperkeratosis, dermal elastic fibers, and type I/III collagen ratio. Results: All patients reported vulvar pruritus at baseline. After treatment, 50% became asymptomatic (p = 0.016), with a reduction in mean VAS score from 8 to 1 (p = 0.016). Burning sensation, present in 71.4% at baseline, decreased to 28.5% at follow-up (p = 0.031), with VAS scores declining from 6 to 0 (p = 0.008). Clinical improvement assessed by GAIS was observed in up to 64.3% of patients, with high inter-rater agreement (ICC = 0.855). Histological regression occurred in 42.9% of cases (p = 0.031). Quality of life significantly improved across all Skindex-29 domains (p < 0.05). There was a reduction in hyperkeratosis (p = 0.032) and in type I/III collagen ratio (p = 0.022), along with reappearance of dermal elastic fibers (p = 0.024). Conclusion: CO₂ laser therapy reduced symptoms and improved clinical, histological, and quality-of-life outcomes, suggesting therapeutic potential for VLS. Larger controlled studies are warranted.

## Introduction

Lichen scleros*u*s (LS) is a chronic inflammatory skin disease that primarily affects the anogenital region of women [1]. The main symptoms are itching, vulvar burning, and dyspareunia, impacting health-related quality of life (HRQoL) [2, 3]. In addition, women diagnosed with vulvar lichen sclerosus (VLS) require disease remission because it has a significant potential for destroying the architecture of the external genitalia and can also be associated with squamous cell carcinoma of the vulva [1, 3, 4].

VLS has its presentation as white patches or plaques, usually symmetrically distributed, on the labia minora and labia majora. These plaques can encircle the vulva, the perineum and the perianal region, displaying a typical figure of an eight [5, 6]. The epidermis may appear in three different forms: Atrophic, normal, or hypertrophic when observed under the microscope [6–8]. Typical histological LS findings include compact ortho-hyperkeratosis, follicular tamponade, hydropic or vacuolar degeneration of basal cells, decreased melanocytes and loss of dermal papillae. In addition, homogenization and hyalinization of collagen in the superficial dermis, and a dense underlying chronic inflammatory infiltrate in the form of a band can be also seen [1, 2–4, 6, 9]. Another histological finding is the disappearance of elastic fibers in the papillary dermis [8, 10].

From an etiopathogenic perspective, although not yet fully understood, evidence suggests two main pathogenic events that can occur because of a genetic susceptibility [11, 12] and environmental triggers [5, 13, 14]. These events are the following: (a) Changes in immune reactivity including autoimmunity and chronic inflammation, and (b) Promotion of fibroblast growth and activity, associated with abnormal collagen synthesis and progressive formation of hyalinized and sclerotic dermal tissue [13, 15, 16]. It is known that the formation of sclerotic tissue in LS involves multiple pathways [1, 17, 18] and it is speculated that collagen increase observed in cases of VLS may reflect poor immunogenic tissue repair response to the disappearance of elastic fibers in the superficial dermis [19].

There is no cure for VLS and current conventional treatment is based on topical application of potent or ultra-potent corticosteroids [1, 2, 4]. The chronic nature of VLS is one of its main problematic characteristics, because relapses are inevitable when treatments are discontinued [13]. In addition, there is a subgroup of patients presenting more severe and hyperkeratotic condition, in which VLS remains recalcitrant despite treatment [20, 21]. Unfortunately, clinicians have limited strategies for managing these patients. Therefore emerging treatments, including energy-based modalities such as fractional and microablative carbon dioxide (CO2) laser (LCO2) have been studied [20, 23–25].

The objective of this study was to evaluate the clinical and histological response of patients with VLS undergoing CO2 laser therapy.

## Materials and methods

### Study design

This is an interventional, uncontrolled, unblinded, prospective, quasi-experimental clinical pre-post intervention type study. It was conducted between April 2021 and April 2025 in one of the Gynecology outpatient clinics at a university hospital. This study was registered on December 4, 2021, in the Brazilian Registry of Clinical Trials (ReBEC), under number RBR-54ymm8f.

### Patient selection 

Since this was a feasibility study, with a comprehensive clinical-histological analysis, focused on a rare disease, convenience sampling was used. The study involved 14 patients with a clinical and histological diagnosis of VLS, who met the inclusion criteria. 

#### Inclusion criteria 

Patients over 18 years old diagnosed with symptomatic VLS, who have never received prior treatment for VLS (“treatment-naive”) or who have not responded adequately to high-potency corticosteroid therapy (“VLS refractory to corticosteroid therapy”) were recruited. For inclusion in this study, patients with VLS refractory to corticosteroid therapy who were still using topical corticosteroids had to discontinue medication for at least four weeks prior to the initial assessment.

#### Exclusion criteria

Patients were excluded from the study based on the following criteria: Absence of “hyalinized band” in the superficial dermis of the initial biopsy; presence of neoplasia, history of tendency to keloids; genital inflammatory and/or infectious diseases at the time of recruitment; presence of immunosuppression; history of previous vulvar radiotherapy; gynecological surgeries within the previous six months; use of topical therapy within four weeks prior to the study participation inclusion.

### Intervention protocol

LCO2 (SmartXide2 V2LR CO2 laser system, MonaLisa Touch, DEKA, Florence, Italy) was used. A topical anesthetic based on 4% lidocaine (Dermomax 40 mg/g; Aché Laboratórios Farmacêuticos S. A., Guarulhos, Brazil) was applied 40 to 60 min prior to laser session. The laser protocol consisted of three sessions, with an average interval of 30 days, using the following settings: Power 24 W, scan time 400 ms, spot spacing 400u, and stack parameter 1 [26]. The straight probe was used.

### Data collection/study outcomes

The patients selected for the study underwent a gynecological clinical assessment and biopsy of the area(s) of LS that had the most significant changes prior to the proposed treatment. The vulvar biopsy was performed via dermatological puncture. The gynecological clinical assessment consisted in measuring symptoms related to VLS, assessing patients’ quality of life (QoL), and observing the disease physical signs during physical examination of the external genitalia, recorded by photographic images. Patients attended follow up six weeks after treatment completion with a biopsy of the worst area detected and a new clinical examination.

#### Primary outcomes

##### Visual analogue scale for symptoms

A visual analog scale was used to rate three symptoms (itching, burning or stinging sensation, and dyspareunia). Each symptom received a score of 0–10, with 0 being no symptom and 10 being the worst imaginable intensity.

##### Physical examination findings by analysis of vulva photographic records

The patients were examined and photographic documentation was performed by the same researcher at initial and follow up assessments. The comparative photographic analysis was performed by three different medical practitioners, two gynecologists and one dermatologist, using a validated scale for overall aesthetic improvement analysis, called the Global Aesthetic Improvement Scale (GAIS), whose score range from 1 (optimal aesthetic result) to 5 (final aesthetic worse than the initial).

##### General histopathological evaluation

Skin samples were fixed in 10% buffered formalin and sent to the Pathology Laboratory of the university hospital, where they were embedded in paraffin. 5-micron thick sections were obtained. The material was stained with hematoxylin/eosin (HE), and the VLS histological diagnosis was made in the initial anatomopathological study, using as minimum criteria the presence of the standard vacuolar interface reaction associated with the hyalinized band of collagen in the superficial dermis / dermal sclerosis of any thickness, located between the inflammatory infiltrate and the epithelium or around the vessel walls. At the final biopsy, the same criteria were used to analyze the overall histological response. If there was regression of initial findings, the outcomes were considered positive. If histological findings were unaltered or worsened, the outcomes were considered negative.

#### Secondary outcomes

##### QOL assessment

To measure QoL, the Portuguese (Brazilian) version of the North American self-administered Skindex-29 questionnaire was used [27]. All responses were transformed into a scale of 0 to 100: 0 for a positive effect on the patient’s QoL and 100 for a negative effect.

##### Specific histopathological evaluations

In addition to being stained with HE, the skin samples from the study were sent for processing and the following special stains were empoloyed: Verhoeff, Masson’s Trichrome, Picrosirius and Fontana Masson [28, 29]. In HE staining, the layers of the epidermis (stratum corneum, granulosum, spinosum, and basale) were evaluated in all samples, as well as the presence and intensity of lymphocytic infiltrate, and the thickness of sclerosis was measured. The presence of collagen fibers in the connective tissue was identified by special Masson’s trichrome and Picrosirius staining. With the aid of polarized lens, type I collagen fibers were showed by an orange-red birefringence pattern, and type III collagen fibers by a yellow-green birefringence pattern. With the aim of demonstrating the elastic fibers, Verhoeff staining was used to stain these fibers by black. The Fontana Masson method was used to demonstrate the presence of melanin, an argentaffin substance. Finally, Image J software was also used through histomorphometric techniques. This software quantified the expression of collagens I and III and the elastic fibers present in the superficial dermis, and also assisted in measuring the thickness of sclerosis in Masson’s trichrome staining [30].

### Statistical analysis 

IBM SPSS 22v software was used for data storage and statistical analysis. The study results were summarized in absolute and percentage values, mean, median, standard deviation, minimum and maximum values. When the data had a normal distribution, they were represented by mean and standard deviation, while for ordinal or non-parametric distribution data, median, minimum, and maximum were used [31]. For the change analysis (before vs. after), we used the McNemar test for dichotomous variables and the Wilcoxon test for ordinal data, both using exact statistics [32]. The significance level adopted was p < 0.05 [33].

## Results

Of the 27 patients who had met the inclusion criteria for this study, 12 were excluded after initial histological analysis due to the absence of the characteristic band of hyalinization in the superficial dermis. One patient withdrew from the study. Thus, a total of 14 patients with VLS participated in this study. There was no drop-out on the follow-up or deviations from the protocol.

The participants age average was 57 years (SD ± 11.31) and only about a third of them were sexually active (28.6%), which made it impossible for dyspareunia to be assessed. The majority (71.4%) considered themselves to be of Caucasian ethnicity. Most women were also postmenopausal (71.4%) and a minority of these (30%) were using hormone therapies. The median duration of illness was 24 months, but only four patients (28.6%) had a history of previous treatment for LS. Only two participants (14.2%) had no clinical comorbidities. The main comorbidities among our patients were dyslipidemia, hypertension, glycemic alterations (diabetes or pre-diabetes) and hypothyroidism. Finally, three participants (21.4%) had family members with LS.

The main clinical outcomes are shown in Table 1 including significant improvement of the parameters analyzed after treatment.

**Table 1:** Clinical outcomes assessed before and after laser therapy (*n* = 14)

Clinical outcomes	Pre-treatment	Post-treatment	*P* value
Presence of vulvar pruritus, n (%)	14 (100)	7 (50)	*0.016*
Presence of vulvar burning/stinging, n (%)	10 (71.4)	4 (28.5)	*0.031*
VAS for pruritus, median (minimum – maximum)	8 (3–10)	1 (0–8)	*0.016*
VAS for vulvar burning, median (minimum – maximum)	6 (0–10)	0 (0–6)	*0.008*
Skindex Total, mean (SD)	49.36 (14.50)	31.82 (12.97)	*0.004*
Skindex emotional domain, mean (SD)	57.72 (19.66)	38.00 (18.44)	*0.008*
Skindex Psychosocial domain, mean (SD)	36.78 (19.22)	23.68 (11.53)	*0.009*
Skindex symptom domain, mean (SD)	59.4 (11.17)	36.74 (17.00)	*0.001*
Change in vulvar skin texture, n (%)	14 (100)	6 (42.9)	*0.031*

*VAS* Visual analog scale, *SD* Standard deviation

**Fig. 1:**
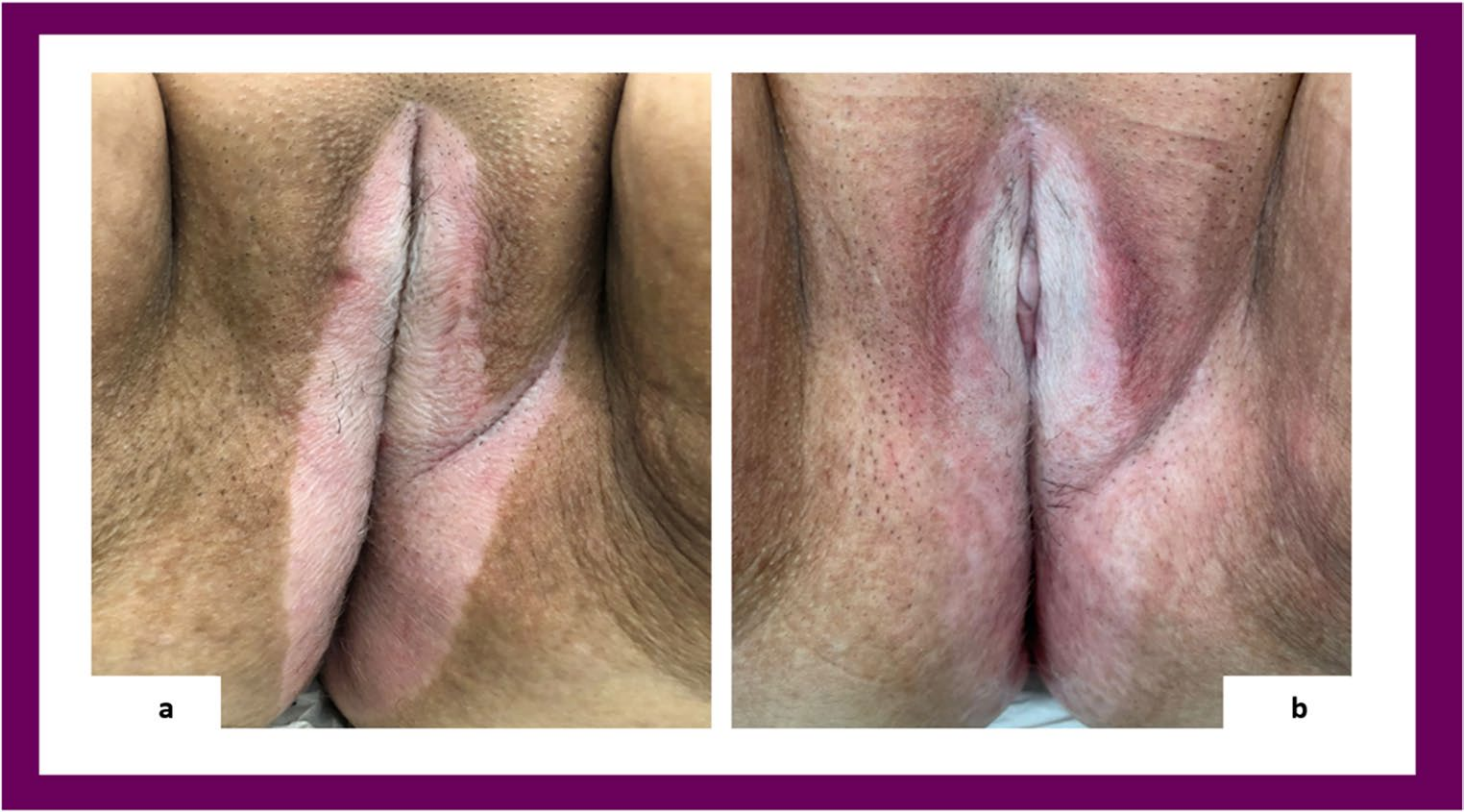
Example of a patient showing overall improvement of the visual appearance of the lichen sclerosus on the GAIS score. **a** shows an extensive area of hypochromia involving the periclitorian region, labia minora and labia majora, perineal and perianal regions, extending to the left genito-crural sulcus, where an extensive fissure is observed; textural changes can also be seen, with atrophy resembling “cigarette paper.” **b** shows the same patient six weeks after laser treatment completion. At that point it is possible to observe improvement on the skin texture and on the extent of hypochromia showing areas of repigmentation

There was overall improvement in VLS on the GAIS index (score ≤ 3) for nine patients (64.3%), eight patients (57.5%), and seven patients (50%), respectively, according to the analysis of the dermatologist and the two gynecologist examiners. A great deal of consensus was also found among the three examiners based on the intraclass correlation coefficient (ICC: 0.855). Figure 1 shows one patient who presented with significant visual improvement, whose GAIS score was 2 (marked improvement, but not complete) presented by the three study examiners.

**Fig. 2:**
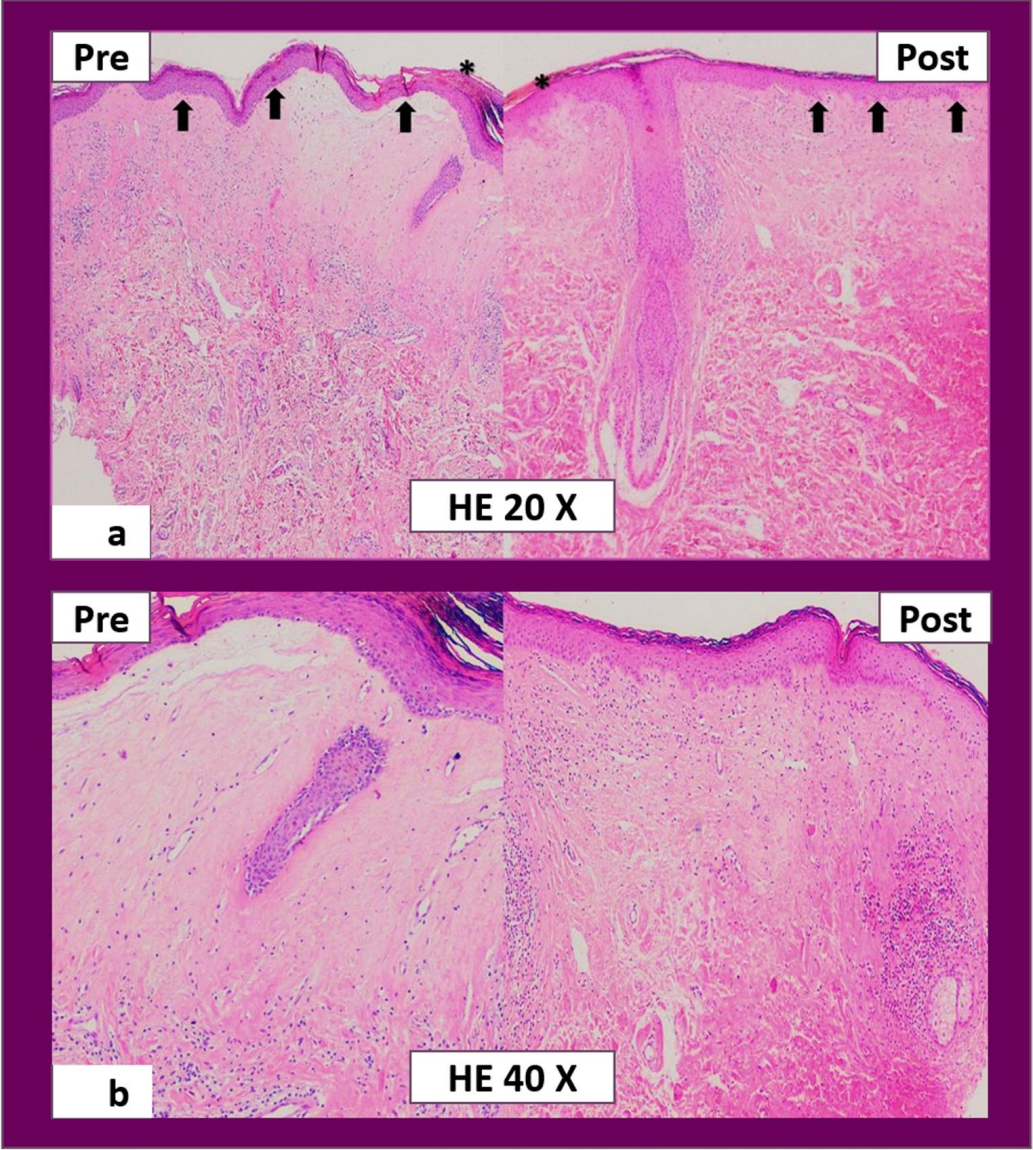
Example of a patient presenting overall histological improvement of lichen sclerosus after laser therapy. **a** - In the epidermis, there is a compact and moderate eosinophilic thickening in the stratum corneum in the pre-treatment phase, which was reduced by half in the post-treatment phase (asterisks). In addition, an interpapillary ridge contour is observed after treatment (arrows). **b** - At higher magnification, lymphocyte attack on the basal layer of the epidermis and vacuolar degeneration of basal keratinocytes were observed, which had not occurred in post-treatment phase. In the papillary dermis, more sclerosis, edema, and lymphocytic infiltrate were observed in the pre-treatment phase compared to the post-treatment phase

Regarding histopathological outcomes (Table 2), signs of LS remittance were observed in six women (42.9%) at the final biopsy (*p* = 0.031). Figure 2 shows an example of histological improvement in standard HE staining.

**Fig. 3:**
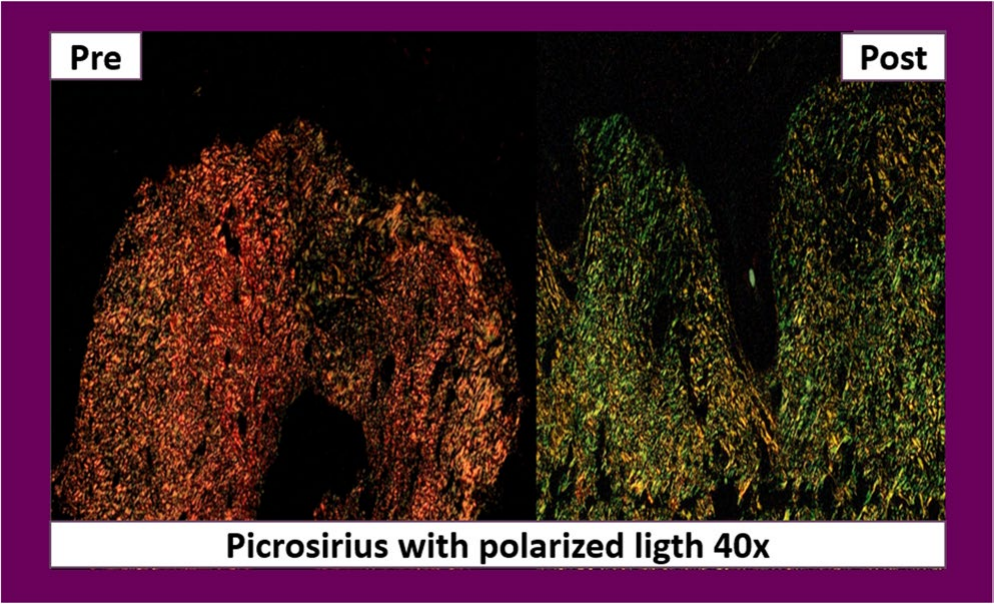
Change in the predominance of collagen type after laser therapy. In the pre-treatment image (left), the predominance of type I collagen (red-orange) is evident, with a reversal of the collagen fiber pattern to type III collagen (green) in the post-treatment image (right)

Regarding specific histological parameters, three showed statistically significant improvement in the morphological evaluation: The stratum corneum, elastic fibers, and collagen fibers (Table 2). There was also a change observed in the pattern of collagen fiber predominance, with a reduction in type I fibers and an increase in type III fibers after treatment (Fig. 3).

**Table 2:** Histological outcomes evaluated before and after laser therapy (*n* = 14)

Histological outcomes	Before laser therapy	After laser therapy	*P* value
Presence of pink stratum corneum with moderate to intense compaction, n (%)	11 (78.5)	6 (42.8)	*0.032*
Presence of corneal plug, n (%)	6 (42.9)	4 (28.6)	*0.433*
Number of cell rows in the granular layer, mean of 5 fields (SD)	4.57 ± 1.83	4.07 ± 1.73	*0.457*
Number of cell rows in the spinous layer, mean of 5 fields (SD)	6.50 ± 2.98	6.21 ± 2.66	*0.798*
Presence of intact basal layer, n (%)	5 (35.7)	9 (64.3)	*0.176*
Absent/scarce pigmentation (melanin) in the basal layer in Fontana Masson, n (%)	10 (71.4)	7 (50.0)	*0.581*
Presence of moderate to intense lymphocytic infiltrate in HE, n (%)	9 (64.3)	6 (42.8)	*0.173*
Moderate to intense elastic fibers in the papillary dermis in Verhoeff, n (%)	4 (28.6)	11 (78.6)	*0.024*
Thickness of sclerosis in HE in microns, mean (SD)	557.1 ± 115.8	521.4 ± 192.9	*0.476*
Thickness of sclerosis in Masson’s trichrome in microns, mean (SD)	542.9 ± 139.8	464.3 ± 173.7	*0.187*
Thickness of sclerosis in Picrosirius in microns, mean (SD)	542.9 ± 150.5	478.6 ± 200.7	*0.338*
Moderate to intense type I collagen in the papillary dermis under polarized light in crosses, n (%)	12 (85.7)	5 (35.7)	*0.004*
Moderate to intense type III collagen in the papillary dermis under polarized light in crosses, n (%)	5 (35.7)	12 (85.7)	*0.009*

*SD* Standard deviation

The results of the digital analysis can be seen in Table 3. No adverse effects were observed during and after laser use, except for mild to moderate local discomfort during application. No patient had to interrupt the procedure due to pain.

**Table 3:** Semi-quantitative digital analysis of the histological outcomes (*n* = 14)

Histological outcomes (digital analysis)	Before laser therapy	After laser therapy		*P* value
Pigmentation (melanin) in the basal layer in Fontana Masson as % of ROI, median (min–max)	0,35 (0–16,8)	1,65 (0–17,1)		*0*,*214*
Presence of elastic fibers in the papillary dermis in Verhoeff as a percentage of ROI, median (min–max)	7,76 (2,65–36,7)	9,3 (2,2–32,7)		*0*,*808*
Thickness of sclerosis in Masson’s trichrome stain in microns, mean (SD)	679,7 (± 428,25)	747,8 (± 292,40)		*0*,*296*
Type I collagen in the papillary dermis in Picrosirius under polarized light as a percentage of ROI, median (min–max)	28 (3–36)	9 (2–25)		*0*,*012*
Type III collagen in the papillary dermis in Picrosirius under polarized light as a percentage of ROI, median (min – max)	15 (7–48)	23 (7–39)	*0*,*124*	
Collagen I/collagen III ratio, median (min – max)	2,26 (0,3–3)	0,43 (0,29–2,78)	*0*,*022*	

*ROI* Region of interest, *SD* Standard deviation

## Discussion

VLS is a chronic and often debilitating condition due to its incapacitating symptoms and functional impact. Standard treatment with high-potency corticosteroids has been shown to be effective in most cases. However, VLS is an incurable disease, requiring maintenance treatment and adherence to long-term therapy. Affected women have presented high rates of recurrence throughout their lifetime [3]. In this context, our study provides data supporting laser therapy considering that LCO2 promoted a significant clinical response, with some important histological changes, among the participants with VLS in this study. Participants demonstrated improvement in symptoms, QoL, and overall physical examination findings. Reduction of hyperkeratosis and promotion of important changes in the dermal extracellular matrix were also observed.

Previous reports have demonstrated that CO2 laser therapy may be an effective form of treatment for patients with recalcitrant genital LS [20–22, 25]. Similar to the study by Filippini et al. [34], our study was not limited to patients refractory to first- line treatment, but these patients had benefited from our study protocol. Women with VLS without a history of adequate treatment had also demonstrated a significant clinical response. Our results were similar to those obtained by Teodoro et al. [35] in which was reported that CO2 laser therapy was successful in achieving remission or a notable reduction in symptoms in a series of ten cases of hyperkeratotic VLS.

At baseline, participants presented significant decrease in overall QoL measured by Skindex-29, particularly for emotional and physical domains (related to symptoms). With regards to the psychosocial aspect, this metric was moderately reduced according to the cut-off points identified by Prinsen et al. [36]. After treatment, there was significant improvement in overall QoL and in all domains of the Skindex-29. Implications on QoL changed to mild, moderate, absent, and mild, respectively, for the total scores and for emotional, physical, and psychosocial aspects.

Our findings are similar to those found by Burkett et al. [23]. These authors compared 27 patients treated with LCO2 and 24 patients treated with 0.05% clobetasol propionate. Their primary outcome was a change in the mean Skindex-29 score at six months. In the intention-to-treat analysis, the authors observed substantial improvement for the laser group compared to the steroid group, for total score and for Skindex-29 subscores for emotion and symptoms aspects. These authors observed average differences of 16 points on the Skindex-29, associated to a significant change in QOL categories. They observed that the total Skindex-29 scores for emotion and symptoms decreased by more than 16 points in the laser arm, while maximum improvement in the steroid arm was only 6.7 points. In our study, total Skindex-29 scores and scores for emotion, symptoms, and psychosocial aspects decreased by 17.54 points, 19.72 points, 22.66 points, and 13.1 points, respectively. Perhaps the psychosocial sphere is the least impacted because VLS manifests itself in an intimate area, visible only to women’s health professionals and sexual partners, and therefore with restricted exposure, particularly in sexually inactive women, as that was the case of most of our patients.

We observed overall improvement in the appearance of the external genitalia after our laser protocol, seen in 50 to 64.3% of the sample measured by GAIS scale and observed in the photographic documentation. We observed main improvement in texture rather than color restoration, which was only partial, after treatment with LCO2. Salgado et al. [37] conducted a randomized clinical trial to evaluate the feasibility and equivalence of LCO2 compared to standard treatment with 0.05% clobetasol propionate in women with VLS. According to the authors, although the anatomical and morphological characteristics of the vulva did not experience major changes or regression after both treatments, at 12-month follow-up, the LCO2 group showed significant short-term improvement for clinical hyperkeratosis and atrophy, which is consistent with our findings. These authors reported that the improvement observed at short-term follow up may not have remained in the long term because the effects of laser therapy subside over time, and a new rescue session may be necessary.

Regarding the histopathological study, we conducted a comprehensive analysis including morphological and morphometric assessments using standard and special stains aiming to endorse the use of LCO2 in VLS as some of the outcomes investigated showed underlying pathogenic mechanisms.

In a prospective, randomized, double-blind study [38], in which 20 women were assigned to receive LCO2 and 20 women received simulated laser, with a washout period of at least 4 weeks, the primary outcome was improvement in histopathological changes. In the anatomopathological analysis, the researchers quantified the severity of the changes on a scale of 0 to 6 points for 3 items: Loss of epidermal ridges, amount of dermal homogenization, and inflammation. In the intention-to-treat analysis, the histopathology scale score at baseline in the sham group was 4.3, and in the LCO2 treatment group was 4.2 (*p* = 0.89). There was a reduction of 0.2 (improvement) in the histopathology scale score from baseline in the active treatment group (95% CI -1.1, 0.8, *p* = 0.74) and an increase of 0.1 from baseline in the sham treatment group (95% CI -0.90, 1.0, *p* = 0.91), but the change in the histopathological scale score between the active and sham arms was not statistically significant (-0.2; 95% CI -1.14, 1.06, *p* = 0.76). In our study, for the two histological changes also included in our analysis, a trend toward improvement was observed, but without statistical significance. There was a reduction in the inflammatory process (*p* = 0.17) and a reduction in the thickness of dermal homogenization/sclerosis, in microns, for the three stains used: Standard HE (*p* = 0.476), Masson’s trichrome (*p* = 0.187), and Picrosirius (*p* = 0.338). However, we observed the following histological outcomes: Reduction of hyperkeratosis, change in the predominant collagen fiber type, and increase in the amount of elastic fibers.

Regarding the response of the stratum corneum to LCO2, previous studies had reported a reduction in clinical hyperkeratosis, likely allowing for better absorption of topical steroids [6]. In agreement with our histological findings, Teodoro et al. [35], in their series of hyperkeratotic VLS cases, reported a reduction in tissue hyperkeratosis after one to three sessions a month of CO2 laser, with up to two laser passes applied focally to more hypertrophic areas. The authors’ post-treatment histological analysis found trophic epithelium with mild acanthosis and small areas of superficial hyperkeratosis.

In discussions of energy-based treatments, the chosen parameters are fundamental because different power levels, as well as variations in spacing and/or exposure time and/or depth of penetration of the active ingredient, result in different photochemical effects. Since the stratum corneum of the vulvar skin is the first to come into direct contact with the energy delivered by the laser, and considering that VSL frequently manifest as thick plaques of hyperkeratosis, we believe it is important, at least as an initial approach, to focus on greater treatment coverage (density). Therefore, we opted for a smaller spacing of 400 micrometers, which consequently resulted in a larger area irradiated by the laser, and thus we had a statistically significant positive histological response in the stratum corneum of our sample. However, the optimal parameters and number of laser therapy sessions for VSL treatment are not yet defined. More studies are needed, but we hypothesize that these patients may benefit from a greater number of laser sessions, rather than just three sessions, as performed in our protocol. A more extended treatment protocol could be designed with progressive changes in laser settings, shifting from an initial focus on therapy density to a subsequent prioritization of treatment depth.

It is believed that the laser normalizes the cycle of collagenesis and collagenolysis [39, 40] by inducing the breakdown of disorganized collagen fibrils [41], creating more organized collagen bundles and decreasing the thickness and density of the collagen bundle [42]. Bretas et al. [43] contributed to this subject by conducting a study with 14 women symptomatic for postmenopausal genitourinary syndrome, who underwent three monthly CO2 laser sessions. Participants were assessed at baseline and 1 month after discharge through questionnaires on sexual function and urinary incontinence, through the vaginal health index, pH measurement, and vaginal wall biopsy. These authors concluded that the efficacy of LCO2 in the treatment of menopausal genitourinary syndrome may be related to epithelial renewal and connective tissue remodeling, as there was reversal of vaginal atrophy and neocollagenesis. Regarding this last finding, the immunohistochemical study showed an increase in collagen III (with *p* < 0.01), with a decrease in the collagen I: III ratio (*p* = 0.001), while it was observed, similarly to our study, that in the analysis with Picrosirius staining, there was an increase in type III collagen fibers (with *p* < 0.05) and a decrease in the proportion of type I fibers (*p* < 0.05). Collagen III has finer fibers than collagen I, and in the study by Bretas et al. [43], the authors also observed a reorganization of collagen fibers after laser therapy, underscored by the presence of new parallel and homogeneous fibers. On the other hand, few studies have similarly investigated the underlying mechanisms of laser therapy in patients with VLS. Recently, Marzec et al. [44] analyzed changes in gene expression, including collagen genes and heat shock protein, in the VLS tissue of two women three months after LCO2 application. In addition to symptomatic improvement, the authors detected statistically significant changes in the expression of COL1A2 (collagen I), HSPA1A, which translates the heat shock protein HSP 70 − 1, and HSPA1B, which translates the heat shock protein HSP 70 − 2, with an increase in all of these. The increase in type I collagen in response to LCO2, was indicated by these authors but not reproduced in our samples. This may be related to the time between the end of the laser therapy sessions and the biopsy, which was 3 months and 45 days, respectively. For a shorter time for histological reanalysis, we observed higher levels of immature type III collagen, which is progressively replaced by type I collagen—a process that can take months [45].

Our study, therefore, aligns with other studies in the literature that address the mechanism of action of the LCO2 on the skin [43].

Regarding elastic fibers, Marzec et al. [44] also investigated the expression of the ELN gene, which encodes the elastin protein, before and after the CO2 laser protocol. In their research, an increase in the amount of those fibers were observed after the intervention. However, the difference was not statistically significant. In our study, we observed that LCO2 stimulated significant production of elastic fibers as these fibers are commonly reduced in the homogenized superficial dermis of tissues presenting LS [19]. Addressing this issue may be a key point in the management of this disease pathogenesis [19]. Our findings favors the use of laser therapy for VLS cases because this therapy seems to minimize the pathogenic processes of LS dermatosis.

A relevant issue in research on the treatment of VLE concerns the definition of the ideal site for therapeutic control biopsy. Clinically, it is recommended to sample areas with more prominent disease characteristics, prioritizing those with the worst appearance, also for the identification of complications [3, 6]. However, most studies do not explicitly state the criteria for selecting the post-treatment biopsy site. Salgado et al. [37] performed a new biopsy in a region contralateral to the initial one, while Mitchell et al. [38] performed it in an adjacent area. Both methods have limitations, because, despite the typical symmetry of anogenital lesions, their distribution is variable and may include areas of malignant transformation close to less severe lesions [4, 7]. In addition, biopsies very close to the previous one may contain fibrosis, making histological comparisons difficult. Thus, given the lack of consensus in the literature, it was decided to perform a biopsy of the most altered areas before and after treatment, aiming to reduce biases without attributing changes resulting solely from the choice of sampling site to the laser effect.

Another positive point of our study was that the confounding variables had been reduced by our research protocol. Our participants underwent a washout period. Monotherapy was applied throughout the study period. And all participants had been assessed for VLS histopathological diagnosis at baseline.

However, the present study was limited by non-randomized and non-comparative design, limited sample size, lack of mild cases in our sample and short-term follow-up.

Uncontrolled studies have significant limitations in assessing the placebo effect, since they cannot separate the real effect of the intervention from the psychological and non-context-specific effects of the treatment, particularly in relation to subjective data. Therefore, objective measures, such as histopathological changes less influenced by the placebo effect, were included as primary and secondary outcomes in this study.

## Conclusions

In conclusion, our findings support that LCO_2_ in patients with VLS contributed to symptom relief, increase in QoL, and overall aesthetic improvement of external genitalia upon physical examination.

As per the histological response, overall improvement was observed. In addition, collagenesis and elastogenesis increase was noted, incluing a reduction of compact hyperkeratosis. These findings may explain the overall clinical improvement observed.

Preliminary results should be confirmed by randomized controlled trials to enable therapeutic regimen enhancement in order to maximize the clinical and histological response to laser therapy in VLS patients.

## Data Availability

The data that support the findings of this study are available from the corresponding author upon reasonable request.
